# Cereal type and combined xylanase/glucanase supplementation influence the cecal microbiota composition in broilers

**DOI:** 10.1186/s40104-022-00702-6

**Published:** 2022-05-04

**Authors:** Dimitrios Kouzounis, Jannigje G. Kers, Natalia Soares, Hauke Smidt, Mirjam A. Kabel, Henk A. Schols

**Affiliations:** 1 Laboratory of Food Chemistry, Wageningen University & Research, 6708 WG Wageningen, The Netherlands; 2 Laboratory of Microbiology, Wageningen University & Research, 6708 WE Wageningen, The Netherlands; 3 Huvepharma NV, 2600 Berchem, Belgium

**Keywords:** Arabinoxylo-oligosaccharides, Broiler gut microbiota, Cereal non-starch polysaccharides, Feed enzymes, Fiber fermentation, Prebiotics, 16S rRNA

## Abstract

Dietary fiber-degrading enzyme supplementation in broilers aims at off-setting the anti-nutritive effect of non-starch polysaccharides and at promoting broiler health. Recently, we demonstrated that xylanase/glucanase addition in wheat-based diet improved nutrient digestibility, arabinoxylan fermentability and broiler growth. Conversely, maize arabinoxylan was found to be recalcitrant to xylanase action. These findings suggested that enzyme-mediated improvement of nutrient digestion and carbohydrate fermentation depended on the cereal type present in the diet, and may have contributed to broiler growth. Hence, we aimed at further investigating the link between dietary enzymes and carbohydrate fermentation in broilers, by studying the impact of enzyme supplementation in cereal-based diets, to the microbial communities in the ileum and ceca of broilers. For that purpose, 96 one-day-old male broilers were randomly reared in two pens and received either wheat-based or maize-based starter and grower diets. At d 20, the broilers were randomly assigned to one out of four dietary treatments. The broilers received for 8 d the wheat-based or maize-based finisher diet as such (Control treatments; WC, MC) or supplemented with a xylanase/glucanase combination (Enzyme treatments; WE, ME). At d 28, samples from the digestive tract were collected, and the ileal and cecal microbiota composition was determined by 16S ribosomal RNA gene amplicon sequencing. A similar phylogenetic (alpha) diversity was observed among the four treatments, both in the ileal and the cecal samples. Furthermore, a similar microbial composition in the ileum (beta diversity) was observed, with lactobacilli being the predominant community for all treatments. In contrast, both cereal type and enzyme supplementation were found to influence cecal communities. The type of cereal (i.e., wheat or maize) explained 47% of the total variation in microbial composition in the ceca. Further stratifying the analysis per cereal type revealed differences in microbiota composition between WC and WE, but not between MC and ME. Furthermore, the prevalence of beneficial genera, such as *Faecalibacterium* and *Blautia*, in the ceca of broilers fed wheat-based diets coincided with arabinoxylan accumulation. These findings indicated that fermentable arabinoxylan and arabinoxylo-oligosaccharides released by dietary xylanase may play an important role in bacterial metabolism.

**Supplementary Information:**

The online version contains supplementary material available at 10.1186/s40104-022-00702-6.

## Background

The importance of a balanced microbial gut ecology for healthy broilers is widely acknowledged [1–3]. In addition, the interplay between gut microbiota and non-digestible feed components has been described to be crucial for poultry health [2]. Therefore, the controlled steering of gut microbiota through dietary interventions may contribute to improved broiler health and growth [4, 5]. For example, the dietary provision of oligosaccharides exhibiting prebiotic and immunomodulatory properties has been suggested to beneficially impact nutrient digestibility and broiler performance [2, 6]. In addition, oligosaccharide supplementation was accompanied by the proliferation of beneficial microbiota, such as bifidobacteria and lactobacilli, the decrease of pathogenic bacteria, and the pronounced short chain fatty acids (SCFAs) formation in the hindgut [5, 7, 8].

In cereal-based poultry diets, non-starch polysaccharides (NSP) are the major carbon source for fermentation. Arabinoxylan (AX) is the main NSP in cereals, such as wheat and maize [9]. As such, AX is an important substrate for bacterial fermentation and SCFAs production in the broiler hindgut. AX fermentability was shown to depend on the cereal type, with wheat AX being considered more easily fermentable than maize AX [10–12]. At the same time, soluble AX can limit nutrient digestibility and promote pathogen growth in the broiler small intestine [13, 14]. In addition, insoluble AX may contribute to nutrient encapsulation by the cereal cell wall matrix, and consequently, to decreased digestibility [13]. Feed supplementation with NSP-active enzymes (NSPases), such as xylanase and glucanase, has been shown to offset detrimental effects of AX on broiler health [3, 13]. For instance, xylanase supplementation in wheat-based diets decreased digesta viscosity, and coincided with increased nutrient digestibility and animal performance [10, 14, 15]. Moreover, xylanase improved SCFAs formation similarly to the direct arabinoxylo- and xylo-oligosaccharides (AXOS, XOS) supplementation [7, 16]. It has been previously shown that both AXOS and XOS exert prebiotic properties in vitro when using human fecal samples as inoculum [12, 17–19]. Recently, we demonstrated that xylanase released AXOS and XOS in vivo, in the broiler gut [11]. It is, therefore, hypothesized that NSPases can modulate gut microbiota by the provision of fermentable oligosaccharides in the ceca.

Understanding the interaction between dietary components and intestinal microbiota is necessary to optimize NSP utilization, in order to promote broiler growth. Therefore, we investigated for wheat-based and maize-based diets the influence of combined xylanase and glucanase supplementation, on the ileal and cecal microbial communities in broilers, by employing 16S rRNA gene amplicon sequencing.

## Materials and methods

### Experimental design and dietary treatments

The work discussed here is part of a larger study on NSP utilization in broilers described in detail elsewhere [11]. The study was conducted at the facilities of the Laboratory for Animal Nutrition and Animal Product Quality (LANUPRO), Department of Animal Sciences and Aquatic Ecology, Ghent University (Belgium), in accordance with the ethical standards and recommendations for accommodation and care of laboratory animals covered by the European Directive 2010/63/EU on the protection of animals used for scientific purposes and the Belgian Royal Decree KB29.05.13 on the use of animals for experimental studies. In brief, 96 one-day old male broilers (Ross 308) (Vervaeke-Belavi, Tielt, Belgium) were randomly assigned to two separate pens and were fed ad libitum either with wheat-soy or maize-soy starter feed (d 0–10) and grower feed (d 10–20) diets, provided in mash form (Table 1). At d 20 the birds were allocated according to body weight to pens with a wire floor. Four dietary treatments; Wheat Control (WC), Wheat Enzyme (WE), Maize Control (MC) and Maize Enzyme (ME) were assigned to each pen following a randomized block design, with the blocking factor referring to the spatial organization in the facility. Each dietary treatment consisted of six replicate pens, with four birds per pen. The broilers had ad libitum access to the finisher feed diet, being fed as such (Control diets) or supplemented with (Enzyme diets) commercial endo-xylanase and endo-glucanase preparation (powder form) from *Trichoderma* spp. (Huvepharma NV, Berchem, Belgium) (Table 1), as prepared by Research Diet Services B.V. (Wijk bij Duurstede, The Netherlands). The birds were weighed after an adaptation period (d 20–24), and then continued to be fed finisher diets until d 28. Feed intake was measured daily per pen (d 25–28). During this period, excreta were collected twice daily, homogenized, and an aliquot of a minimum of 250 g fresh material per pen was immediately stored at  −20 °C. At d 28, all birds were weighed, euthanized by cervical dislocation, and the ileum and ceca contents were collected, pooled per pen, and frozen at −20 °C.

**Table 1:** Diet composition of wheat-based and maize-based diets. The data were previously determined and are reported elsewhere [11]

Ingredient, %	Wheat-based			Maize-based		
	Starter	Grower	Finisher	Starter	Grower	Finisher
Wheat	49.4	58.8	65.9	–	–	–
Maize	10.0	5.0	–	57.3	59.6	59.1
Soybean meal 48CP^1^	24.4	19.5	17.0	27.2	24.3	24.3
Toasted soybeans	10.0	10.0	8.0	10.0	10.0	8.0
Soybean oil	1.4	2.4	4.3	0.6	1.7	3.9
Monocalcium phosphate	1.4	1.3	1.0	1.5	1.4	1.2
Limestone	1.4	1.3	1.1	1.4	1.2	1.1
DL-Methionine	0.4	0.3	0.2	0.4	0.3	0.3
L-Lysine HCl	0.3	0.3	0.3	0.3	0.3	0.2
Salt	0.2	0.2	0.3	0.2	0.2	0.3
Na-Bicarbonate	0.3	0.3	0.2	0.3	0.3	0.2
L-Threonine	0.2	0.1	0.1	0.2	0.1	0.1
L-Valine	0.1	0.1	0.1	0.2	0.1	0.0
Coccidiostat	Sacox^2^	Sacox	–	Sacox	Sacox	–
Premix article^3^	0.5	0.5	0.5	0.5	0.5	0.5
Diamol^4^	–	–	1.0	–	–	1.0
Total	100.0	100.0	100.0	100.0	100.0	100.0
Calculated chemical composition, % as is						
ME^5^, MJ/kg	11.8	12.1	12.5	12.0	12.4	12.8
Crude protein	21.8	20.1	18.5	21.2	19.9	19.2
NDF	10.0	10.1	10.0	9.7	9.7	9.4
Crude fat	4.9	5.7	7.1	5.1	6.3	8.0
Arg	1.46	1.31	1.18	1.47	1.37	1.31
Met + Cys	0.69	0.64	0.60	0.67	0.64	0.62
Ile	0.92	0.84	0.76	0.92	0.86	0.83
Leu	1.65	1.49	1.35	1.83	1.74	1.68
Lys	1.14	1.01	0.90	1.18	1.09	1.05
Thr	0.79	0.72	0.65	0.82	0.77	0.74
Val	1.02	0.93	0.85	1.02	0.96	0.92
Ca	0.87	0.81	0.67	0.88	0.78	0.71
Cl	0.16	0.16	0.22	0.16	0.16	0.21
K	0.92	0.83	0.75	0.95	0.90	0.86
Na	0.17	0.17	0.18	0.16	0.16	0.17
Total P	0.68	0.65	0.56	0.70	0.66	0.61
Available P	0.34	0.32	0.27	0.34	0.32	0.29
Analyzed chemical composition, % dry matter						
Dry matter, % as is	–	–	90.3	–	–	89.5
Starch	–	–	40.4	–	–	37.4
Crude protein (N × 6.25)	–	–	20.5	–	–	20.7
Ash	–	–	5.9	–	–	6.5
NSP^6^	–	–	21.0	–	–	18.6
NGP^7^	–	–	9.8	–	–	8.7
AX^8^	–	–	5.0	–	–	3.4
Analyzed enzyme activity (of enzyme-supplemented diets)						
Xylanase, EPU^9^/kg feed	–	–	1550	–	–	1740
Cellulase, CU^10^/kg feed	–	–	240	–	–	190

^1^ *CP*: Crude protein

^2^ Provided by Huvepharma NV, Berchem, Belgium

^3^ Providing per kg of diet: vitamin A (retinyl acetate), 10,000 IU; vitamin D_3_ (cholecalciferol), 2500 IU; vitamin E (dl-α-tocopherol acetate), 50 mg; vitamin K_3_ (menadione), 1.5 mg; vitamin B_1_ (thiamine), 2.0 mg; vitamin B_2_ (riboflavin), 7.5 mg; niacin, 35 mg; D-pantothenic acid, 12 mg; vitamin B_6_ (pyridoxine-HCl), 3.5 mg; vitamin B_12_ (cyanocobalamine), 20 μg; folic acid, 1.0 mg; biotin, 0.2 mg; choline chloride, 460 mg; Fe (FeSO_4_·H_2_O), 80 mg; Cu (CuSO_4_·5H_2_O), 12 mg; Zn (ZnO), 60 mg; Mn (MnO), 85 mg; I (Ca (IO_3_)_2_), 0.8 mg; Co (Co_2_CO_3_(OH)_2_), 0.77 mg; Se (Na_2_O_3_Se), 0.15 mg

^4^Used as acid insoluble ash (AIA) digestibility marker (Franz Bertram GmbH, Hamburg, Germany)

^5^Metabolizable energy

^6^Non-starch polysaccharides; calculated as the difference between total carbohydrates and starch

^7^Non-glucosyl NSP; calculated as the sum of sum of arabinosyl, xylosyl, galactosyl, uronyl, mannosyl, rhamnosyl and fucosyl units

^8^Arabinoxylan; calculated as the sum of arabinosyl and xylosyl units

^9^Amount of enzyme which releases 0.0083 μmol of reducing sugars (xylose equivalents) per minute from oat spelt xylan at pH 4.7 and 50 °C

^10^Amount of enzyme which releases 0.128 μmol of reducing sugars (glucose equivalents) per minute from barley β-glucan at pH 4.5 and 30 °C

### DNA extraction

DNA was extracted, from 0.25 g pooled ileal or cecal content, using 700 μL Stool Transport and Recovery (STAR) buffer (Roche Diagnostics Nederland BV, Almere, the Netherlands), as described in detail before [20]. DNA concentration was measured with a NanoDrop ND-1000 spectrophotometer (NanoDrop® Technologies, Wilmington, DE, USA), and DNA was stored at −20 °C until further use. Extracted DNA was diluted to 20 ng/μL in nuclease free H_2_O. All PCR plastics were UV irradiated for 15 min before use.

### Microbiota analysis

For 16S rRNA gene amplicon sequencing, barcoded amplicons covering the variable regions V4 of the 16S rRNA gene were generated by PCR using the 515F and 806Rd primers. The samples were amplified in duplicate using Phusion hot start II high fidelity polymerase (Finnzymes, Espoo, Finland) and checked for correct size and concentration. The PCR reactions contained 36.5 μL nucleotide free water (Promega, Madison, WI, USA), 0.5 μL of 2 U/μL polymerase, 10 μL of 5 × HF buffer, 1 μL of 10 μmol/L stock solutions of each of the forward and reverse primers, 1 μL 10 mmol/L dNTPs (Promega) and 1 μL template DNA. Reactions were held at 98 °C for 30 s and amplification proceeded for 25 cycles at 98 °C for 10 s, 42 °C for 10 s, 72 °C for 10 s and a final extension of 7 min at 72 °C. Two out of the 24 ileal samples contained a low amount of DNA (< 1 ng/μL) and did not pass our quality control. Synthetic mock communities of known composition were added as positive controls [21], and samples with nuclease free water were added as no-template negative controls to ensure high quality sequencing data. The samples were sent to Eurofins Genomics Germany GmbH (Ebersberg, Germany) for sequencing on an Illumina Hiseq2500 instrument. Data was analyzed using NG-Tax 2.0 [22]. De novo amplicon sequence variants (ASVs) were generated, using an abundance threshold of 0.1% on a per-sample basis. Taxonomy was assigned using SILVA 132 16S rRNA gene reference database [23]. The ASVs associated with the family Mitochondria (*n* = 2) and the order Chloroplasts (*n* = 2) were removed from the data for all sequenced samples.

### Chemical analyses

The experiments described in this section were previously performed, and are thoroughly described in our recent publication [11]. Therefore, they are only briefly mentioned here. The dry matter and crude protein contents of diets, ileal digesta and excreta was determined according to the AOAC 935.29 and 990.03 method, respectively, while the dry matter content of cecal samples was determined separately [11]. Ash, and acid insoluble ash (AIA) contents were determined in diets, ileal digesta and excreta [11]. Starch content of the diets was determined according to AOAC 996.11 method. Sugar composition and content of finisher diets and digesta was determined by gas chromatography (neutral sugars) and by the colorimetric *m*-hydroxyphenyl assay (uronic acids) with an automated analyzer (Skalar Analytical B.V., Breda, The Netherlands) [24–26]. SCFAs (acetate, butyrate, propionate, isobutyrate and isovalerate) content in the ceca was determined by gas chromatography [27].

### Calculations

Non-starch polysaccharides content was calculated as the difference between total carbohydrates and starch content. The apparent ileal digestibility (AID) of organic matter (OM = DM – Ash), as well as the apparent total tract recovery (Rec) of arabinoxylan (AX: sum of arabinosyl and xylosyl constituents) and non-glucosyl NSP (NGP: sum of arabinosyl, xylosyl, galactosyl, uronyl, mannosyl, rhamnosyl and fucosyl constituents) were determined using acid insoluble ash (AIA) as digestibility marker [11].

### Statistical analysis

The analysis of the 16S rRNA gene sequence data was carried out using R version 4.0.2. Alpha diversity was determined using phylogenetic diversity and tested with a Kruskal-Wallis test. Pairwise comparisons were tested using a Wilcoxon rank-sum test. Beta diversity was determined using Jaccard-, Bray-Curtis-, unweighted UniFrac (UF)- and weighted UniFrac (WUF)- metrics. Non-parametric permutational analysis of variance (PERMANOVA) tests were used to analyze group differences within multivariate community data. Multivariate microbiota data were visualized using principal coordinates analysis (PCoA). To test for differences in relative abundance of individual genera between two groups, a Wilcoxon rank-sum test was used, and corrected for multiple testing with the Benjamini-Hochberg (BH) procedure. Weighted UniFrac distance-based redundancy analysis (WUF-db-RDA), a multivariate canonical ordination analysis method that takes the phylogenetic makeup of microbial communities into consideration, was performed using ASV level data and other measured parameters [28]. The parameters included as variables were the broiler body weight (BW, d 28), AX total tract recovery (AX_Trec), NGP (NGP_Cec) and AX (AX_Cec) content (% w/w, dry matter basis) in the ceca, SCFAs (acetate, butyrate and propionate content (μmol/g, dry matter basis) in the ceca (Additional File 1: Table S1). Values for these parameters have been reported earlier [11].

## Results and discussion

In our previous work, we investigated the impact of enzyme supplementation in wheat-based and maize-based diets on nutrient digestibility, NSP fermentability and broiler growth [11], and results have been summarized in Table 2. Addition of a commercial preparation containing xylanase and glucanase in the wheat-based diet (WE) was found to improve the apparent ileal digestibility of organic matter compared to the control diet (WC). In addition, dietary xylanase in WE was shown to release AXOS in vivo*,* in the upper gastrointestinal tract (GIT) [11]. Simultaneously, enzyme action in WE improved AX fermentability to SCFAs in the ceca, while it decreased the total tract recovery of AX compared to WC. Conversely, no direct effect of glucanase on carbohydrate fermentation in the ceca could be discerned. The observed improvement in nutrient digestibility and NSP fermentability by enzyme supplementation coincided with higher body weight values in WE compared to WC (Table 2). In contrast, enzyme addition in the maize-based diet (ME) was not found to impact NSP fermentability or nutrient digestibility [11]. These findings were in line with previous research [10, 15], and suggested that AX fermentation proceeded differently in the ceca of broilers fed wheat-based and maize-based diets. Consequently, the cereal type in the diet was expected to affect the impact of xylanase on AX fermentability. Therefore, continuing our efforts to further substantiate the link between dietary NSPases and NSP fermentation in broilers we now employed next-generation 16S rRNA gene amplicon sequencing to examine the potential impact of cereal type and xylanase/glucanase supplementation, on the ileal and cecal microbiota composition of broilers.

**Table 2:** Effect of diet and enzyme supplementation on broiler growth, nutrient digestibility and NSP fermentability [11]

Dietary treatment	BW^1^, g	FCR^2^, g/g	OM-AID, %	AX Rec, %	NGP Rec, %	SCFAs^3^, μmol/g
WC	1290.00^#b^	1.62^#^	72.17^b^	84.78^b^	68.82^b^	239.69^c^
WE	1370.00^a^	1.50	75.31^a^	76.96^b^	68.00^b^	338.03^b^
MC	1311.67^ab^	1.53	74.60^a^	129.29^a^	85.96^a^	472.42^a^
ME	1353.92^ab^	1.50	74.74^a^	116.95^a^	92.98^a^	378.16^ab^
SEM^4^	18.4 ^#^20.4	0.03 ^#^0.03	0.55	4.25	2.58	24.00
*P*-values^5^	0.029	0.053	0.004	< 0.001	< 0.001	< 0.001
Effect of cereal type (Wheat vs. Maize)						
Wheat	1333.64^#^	1.55^#^	73.7	80.9	68.4	288.9
Maize	1332.79	1.51	74.7	123.1	89.5	425.3
SEM	16.0 ^#^16.7	0.02 ^#^0.02	0.51	3.28	1.91	22.2
*P*-values	0.990	0.275	0.210	< 0.001	< 0.001	< 0.001
Effect of enzyme supplementation (Control vs. Enzyme; stratified per cereal type)						
Wheat (WC vs. WE)						
SEM	18.80 ^#^20.60	0.03 ^#^0.03	0.34	2.58	1.63	1.67
*P*-values	0.021	0.018	< 0.001	0.064	0.731	0.021
Maize (MC vs. ME)						
SEM	18.22	0.03	0.71	5.47	3.23	1.59
*P*-values	0.136	0.498	0.889	0.149	0.163	0.024

^1^Body weight measured at d28

^2^Feed conversion ratio measured during the finisher period (d 24–28)

^3^Sum of acetate, butyrate, propionate, isobutyrate and isovalerate in the ceca, expressed on dry matter basis

^4^Standard error of the mean (*n* = 6). ^#^In case of missingness, the adjusted SEM value (*n* = 5) is presented

^5^Estimated by one-way ANOVA. The significance threshold was *P* < 0.05. Values within column bearing different lowercase letters as superscripts differ significantly at *P *< 0.05 (Tukey's HSD test)

### The type of cereal and enzyme supplementation influences microbial communities

**Fig. 1:**
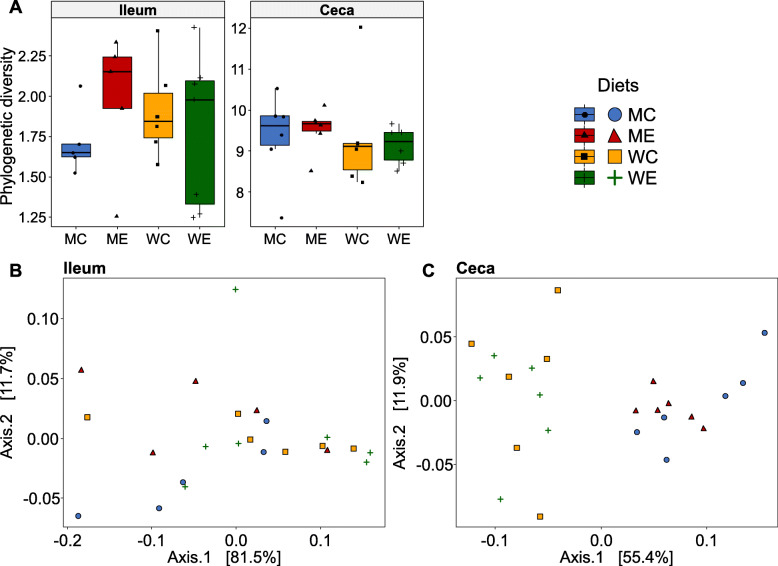
Alpha and beta diversity in the ileum and the ceca across dietary treatments; maize control (MC), maize enzyme (ME), wheat control (WC) and wheat enzyme (WE), (**A**) Phylogenetic diversity (ASV level) across dietary treatments. Whiskers show 95% interval, box 50% interval. Pairwise Wilcoxon rank sum tests (separately for ileum and ceca), corrected for multiple comparisons using the Benjamini-Hochberg procedure showed no difference between groups, (**B**) Principal coordinate plots (PCoA) based on weighted UniFrac distances of ileum samples. **C** Principal coordinate plots (PCoA) based on weighted UniFrac distances of ceca samples

First, phylogenetic diversity, representing biodiversity, was compared across diets and was found to be similar for all diets either in the ileum or in the ceca (Fig. 1A). Other alpha diversity metrics were in agreement and showed similarities among the diets (data not shown). Second, the ileal samples presented similarities in beta diversity across all diets (Fig. 1B). Our findings were in agreement with previous studies showing no effect of cereal type and enzyme supplementation on total anaerobic bacteria and lactobacilli counts [29–31] and on alpha and beta diversity indices in the ileum [10].

**Fig. 2:**
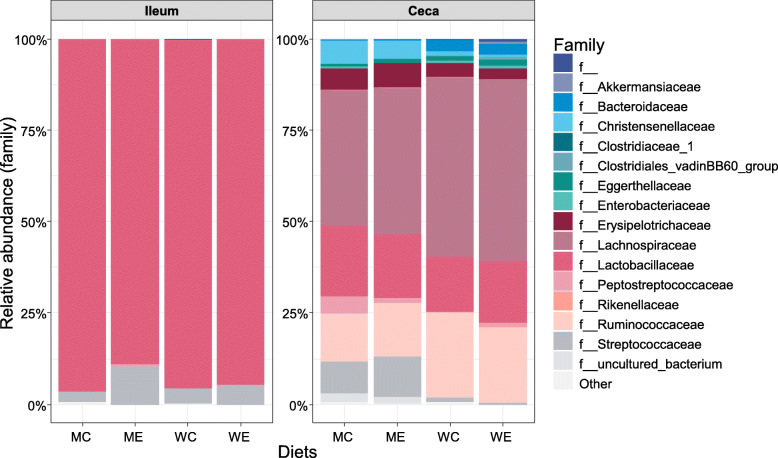
Cumulative relative abundance (%) of microbial taxa at family level. The abundance threshold is 0.01% and each bar represents six samples

The microbial composition was compared at family level in the ileum and ceca across the four diets (Fig. 2). Lactobacillaceae (approximately 90%) and Streptococcaceae (< 10%) were predominant in the ileum. No difference in relative abundance between the diets was observed either at family or genus level. The predominance of Lactobacillaceae in the ileum was in accordance with previous studies [1, 10, 32]. The current findings concur with those stating that lactobacilli in the ileum were not affected by cereal type and xylanase supplementation, with or without addition of glucanase [10, 16, 31]. The ceca are known to harbor a more diverse microbiota composition than the ileum [32, 33]. Indeed, various families were observed in the cecal samples of the wheat-based diets, with members of the family Lachnospiraceae being the main species, followed by members of the families Ruminococaceae and Lactobacilaceae (Fig. 2)*.* The high relative abundance of these families was in line with previous reports [10, 20, 32, 33]. The maize-based diets also presented a diverse microbial ecology in the ceca, though different in composition from the wheat-based diets. For example, members of the families Streptococcaceae, Christensenellaceae, Erysipelotrichaceae and Peptostreptococcaceae presented higher relative abundance, and Bacteroidaceae presented lower relative abundance in the maize-based diets compared to the wheat-based diets (Fig. 2).

The observed effect of the diet on beta diversity corroborated its modulatory influence on cecal microbiota composition (Fig. 1C, Table 3). Weighted UniFrac metrics demonstrated that dietary treatment explained 51.6% (R^2^), and cereal type explained 47.6% of the observed variation between treatments (Table 3). Other beta diversity metrics were in line, but explained a lower proportion of the variation (Table 3). Hence, it was demonstrated that cereal type (wheat vs. maize) in the diet markedly affected the microbial composition in the ceca, regardless of enzyme supplementation. The diet, and the type of cereal in particular, have previously been proposed to influence the broiler intestinal microbiota [34], but experimental evidence is scarce [30, 31, 35]. Nevertheless, a recent study reported increased bifidobacteria counts in the ceca of 35-d old broilers fed with a wheat-based diet compared to a maize-based diet [31]. Additionally, cereal NSP like AX, are known to be utilized as substrates during microbial fermentation to produce SCFAs [2, 36]. Differences in NSP physicochemical properties and inclusion level, as a consequence of their botanical source, may dictate the extent of NSP utilization and, hence, SCFAs formation by microbiota [12, 18]. For instance, the less complex structure and higher water-solubility of wheat AX compared to maize AX [9], are believed to be associated with pronounced cecal fermentation in broilers [10, 11].

**Table 3:** Beta diversity analysis with different distance measures determining microbiota interindividual diversity in the broiler ceca

		Bray-Curtis		Jaccard		Unweighted UniFrac		Weighted UniFrac	
	n	R^2 a^	*P*^*b*^	R^2^	*P*	R^2^	*P*	R^2^	*P*
Dietary treatment	24	0.348	< 0.001	0.281	< 0.001	0.413	< 0.001	0.516	< 0.001
Effect of cereal type									
Wheat vs. Maize	24	0.205	< 0.001	0.162	< 0.001	0.350	< 0.001	0.476	< 0.001
Effect of enzyme supplementation (Control vs. Enzyme; stratified per cereal type)									
Wheat (WC vs. WE)	12	0.276	0.012	0.204	0.014	0.083	0.585	0.064	0.729
Maize (MC vs. ME)	12	0.054	0.765	0.067	0.741	0.115	0.163	0.095	0.351

^a^Percentage of the variation between broilers explained, ^b^*P*-value permutational analysis of variance (PERMANOVA)

It should be noted that enzyme supplementation occurred only during the finisher phase lasting eight days. Therefore, the influence of enzyme supplementation on beta diversity was determined by stratifying the analysis per cereal type (Table 3). No effect of enzyme supplementation during the finisher phase was observed in the maize-based treatments. Conversely, enzyme supplementation for the same period appeared to influence beta diversity in the wheat-based treatments. In particular, the Bray-Curtis and Jaccard distance metrics showed that enzyme supplementation explained 27.6% and 20.4% of the total variation between WC and WE, respectively. However, the corresponding weighted and unweighted UniFrac distance metrics that take phylogenetic relatedness of observed microorganisms into account, did not show significant differences between WC and WE. This suggested that microorganisms of close phylogenetic proximity were predominantly influenced by enzyme supplementation. Overall, NSPase supplementation in the finisher phase exerts a modulatory effect on cecal microbiota, probably related to improved AX fermentability [10, 11, 37].

### Distinct cecal microbial communities coincide with arabinoxylan fermentation

**Fig. 3:**
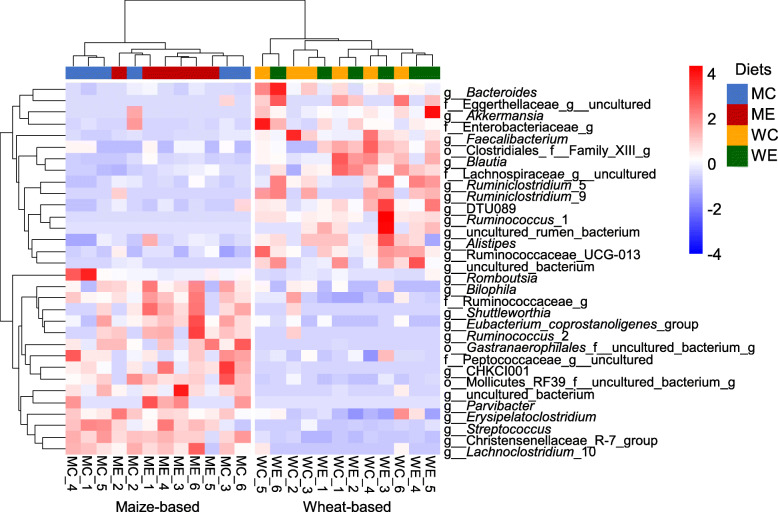
Heatmap of the genera that were significantly different in relative abundance between dietary treatments (Wilcoxon rank-sum test, adjusted *P*-values are corrected for multiple testing using the Benjamini-Hochberg procedure, *P* < 0.05). Each red, white, blue square represents the relative abundance

**Fig. 4:**
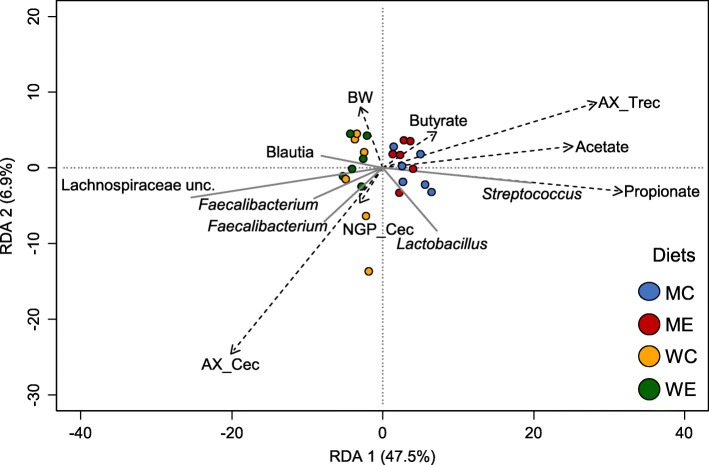
WUF-db-RDA showing the associations between chemical parameters and microbial ASVs. The six best fitting ASV are displayed and samples are colour based on the dietary treatment. The dashed arrows depict broiler body weight (BW, d 28), AX total tract recovery (AX_Trec), NGP (NGP_Cec) and AX (AX_Cec) content (%, w/w) in the ceca and acetate, butyrate and propionate content (μmol/g) in the ceca, and the solid arrows depict the abundance of bacterial groups

The heatmap in Fig. 3 shows all ASV that significantly differed in relative abundance between wheat-based and maize-based treatments. The two hierarchical clusters visualized in Fig. 3 corresponded to either wheat-based or maize-based treatments, further emphasizing the impact of cereal on cecal microbial ecology. In specific, 31 genera were identified as being significantly different, and appeared to distinguish the wheat-based from the maize-based treatments (Fig. 3). Next, body weight, AX, non-glucosyl NSP (NGP) and SCFAs contents in the ceca and AX total tract recovery were added in the WUR-db-RDA model to further disentangle the influence of the diet on cecal microbiota composition (Fig. 4). The top six visualized ASVs separating the diets were classified as one member of an uncultured genus of the family Lachnospiraceae, two ASVs within the genus *Faecalibacterium*, and single ASVs within the genera *Lactobacillus*, *Blautia* and *Streptococcus*.

Members of bacterial taxa such as Lachnospiraceae*, Subdoligranulum*, *Coprococcus, Faecalibacterium* and *Blautia*, all being members of the class Clostridia, are reported to possess an enzymatic arsenal to degrade AX and AXOS [38]. Such genera can be involved in carbohydrate metabolism to produce SCFAs, and may contribute to a healthy gut [2, 39, 40]. Therefore, we hypothesized that an increase in fermentable AX/AXOS in the ceca as a result of xylanase action, present in the NSPase preparation, promoted the proliferation of such microbial communities. To this end, we further explored the potential interrelationships between cecal microbial communities and carbohydrate fermentation patterns using redundancy analysis (Fig. 4). The first dimension (RDA1) explained 47.5% of the total variation and separated the wheat-based from the maize-based diets. RDA2 explained an additional 6.9% of the total variation. MC and ME presented higher total SCFAs (Table 2) and higher individual acetate, butyrate and propionate amounts (μmol/g dry matter) compared to WC and WE (Fig. 4., Additional File 1: Table S1). This observation can partly be explained by different dry matter contents in WC/WE and MC/ME (Additional File 1: Table S1). However, it would have been expected that wheat-based diets would present higher SCFAs values compared to maize-based diets, as a consequence of pronounced NSP fermentability and higher AX content [15, 31]. Both WC and WE presented higher scores for NGP, e.g. AX contents in the ceca than MC and ME, as reported in our recent publication [11]. Therefore, the higher solubility of wheat AX compared to maize AX [9] indicated that more AX entered the ceca and was fermented by the gut microbiota, in the wheat-based diets. The pronounced AX fermentation in wheat-based diets was further documented by the lower AX total tract recovery compared to the maize-based diets (Fig. 4: AX_TRec) [11].

ASVs within *Faecalibactirium*, *Blautia* and Lachnospiraceae were positively associated with AX content in the ceca (Fig. 4). It, therefore, appeared that the proliferation of such genera in the wheat-based treatments coincided with pronounced AX fermentation. It has been previously suggested that fructooligosaccharides (FOS) and long-chain fructans were present in the soluble fraction of ileal digesta of wheat-based diets [11]. FOS and inulin supplementation has been mentioned to also lead to the proliferation of microbiota such as *Bifidobacterium, Lactobacillus, Faecalibacterium* and *Blautia* in both animal and human studies [5, 40, 41]. Consequently, it is expected that both FOS and AX/AXOS present in the wheat-based diets may exhibit bacteria-modulating properties. Still, more research is warranted to determine the relative contribution of different dietary NSP to a healthy gut.

The subtle shift in microbiota composition observed in this study in WE compared to WC (Table 3), could be related to AXOS formation in the proximal GIT by xylanase [11]. The prebiotic potential of AXOS and XOS has been mainly demonstrated by their ability to selectively promote the growth of *Bifidobacterium* and *Lactobacillus* species during in vitro incubations with human fecal inocula [17, 19]. Likewise, supplementation of wheat-based diets with AXOS, XOS and xylanase has been previously shown to exert bifidogenic effect in broilers [7, 42]. Nevertheless, differences in the microbial composition of the human and avian gut [43] may impede the direct extrapolation of AXOS prebiotic potential in poultry. The present findings indicate that AX/AXOS fermentation could be important for the proliferation and function of other beneficial microbiota. So far, *Faecalibacterium* and *Blautia* have been reported to exhibit probiotic properties, while several Clostridiales have been previously associated with improved broiler performance [44, 45]. Therefore, their abundant presence may be highly important for a healthy gut. Although, no direct associations between beneficial microbiota and body weight could be currently established (Fig. 4), it is considered likely that their increased presence may benefit the host. Yet, further research is warranted to unravel potential interactions between dietary enzymes and microbial communities along the GIT, and their impact on animal growth.

## Conclusion

The present study explores for a wheat-based and a maize-based diet the impact of NSPases on the broiler gut microbiota. Findings indicated that the microbial composition in the ceca strongly depended on the cereal type present in the diet. The proliferation of beneficial microbiota such as Lachnospiraceae, *Faecalibacterium* and *Blautia* in the wheat-based treatments compared to the maize-based treatments may be related to the pronounced fermentability of wheat-NSP. In particular, positive associations between fermentable AX content in the ceca and bacterial genera involved in carbohydrate metabolism to SCFAs were observed. These findings further support the importance of AX fermentation for a healthy broiler gut. The present outcomes provide further insight on how the xylanase-mediated AXOS release in vivo improves cecal fermentation and ecology. Notwithstanding, co-fermentation of other dietary NSP next to AX may have proceeded differently in wheat-based and maize-based diets. Consequently, AX fermentability is not expected to be the sole factor explaining the observed differences in microbial composition. The present findings provide important insight on the ability of dietary NSPases to modulate microbial ecology and metabolism in the broiler ceca.

## Supplementary Information

**Additional file 1.**

## Data Availability

The datasets used and/or analysed during the current study are available from the corresponding author on reasonable request.

## Abbreviations

AX: Arabinoxylan
AXOS: Arabinoxylan oligosaccharides
XOS: Xylooligosaccharides
FOS: Fructooligosaccharides
SCFAs: Short-chain fatty acids
NSP: Non-starch polysaccharides
GIT: Gastrointestinal tract
WC: Wheat control diet
WE: Wheat enzyme diet
MC: Maize control diet
ME: Maize enzyme diet
ASV: Amplicon sequence variant
PCoA: Principal Coordinate Analysis
BH: Benjamini-Hochberg
WUF-db-RDA: Weighted UniFrac distance-based redundancy analysis
