# Supplementary material for: Cereal type and combined xylanase/glucanase supplementation influence the cecal microbiota composition in broilers
Source: J Anim Sci Biotechnol. 2022 May 4;13:51. doi: 10.1186/s40104-022-00702-6 (PMC9066912; doi:10.1186/s40104-022-00702-6)
Supplement: Supplementary file 1 — Additional file 1. [file 40104_2022_702_MOESM1_ESM.docx]

Additional File 1

Table S1. Dry matter content (% w/w) (standard deviation), total carbohydrate, constituent arabinosyl (Ara) xylosyl (Xyl) content (% w/w dry matter basis), and acetate, butyrate and propionate content (μmol/g dry matter basis) in the ceca of broilers fed the various dietary treatments. The data were previously determined and are reported elsewhere [1].

| Analyzed parameter  (in ceca) | Diets | | | |
| --- | --- | --- | --- | --- |
|  | Wheat Control (WC) | Wheat Enzyme (WE) | Maize Control (MC) | Maize Enzyme (ME) |
| Dry matter, % w/w | 26.57 (1.71) | 23.12 (1.24) | 18.19 (1.59) | 19.02 (0.54) |
| Carbohydrate content, % w/w dry matter basis | | | | |
| NGP^1^ | 5.22 | 3.88 | 4.14 | 3.81 |
| AX^2^ | 0.89 | 0.52 | 0.32 | 0.27 |
| Short chain fatty acid content, μmol/g dry matter basis^3^ | | | | |
| Acetate | 172.66 | 250.94 | 354.47 | 287.77 |
| Butyrate | 53.12 | 73.08 | 78.46 | 59.29 |
| Propionate | 11.02 | 11.15 | 31.43 | 23.60 |

^1^Non-glucosyl NSP; calculated as the sum of sum of arabinosyl, xylosyl, galactosyl, uronyl, mannosyl, rhamnosyl and fucosyl units

^2^Arabinoxylan; calculated as the sum of arabinosyl and xylosyl units

^3^ANOVA results for 1) WC *vs* WE: Acetate, *P* = 0.014, Butyrate, *P* = 0.044, Propionate, *P* = 0.906, 2) MC *vs* ME: Acetate, *P* = 0.037, Butyrate, *P* = 0.010, Propionate, *P* = 0.039. Significance was set at *P* < 0.05.

**References**

1. Kouzounis D, Hageman JA, Soares N, Michiels J, Schols HA. Impact of xylanase and glucanase on oligosaccharide formation, carbohydrate fermentation patterns, and nutrient utilization in the gastrointestinal tract of broilers. Animals. 2021;11:1285. <https://doi.org/10.3390/ani11051285>
